# Identification and characterization of waterlogging-responsive genes in the parental line of maize hybrid An’nong 876

**DOI:** 10.1590/1678-4685-GMB-2023-0026

**Published:** 2024-01-08

**Authors:** Hongying Wu, Haitao Yu, Xingen Zhang, Yixiao Wang, Hongjia Zhu, Yang Zhao, Qing Ma

**Affiliations:** 1 Anhui Agricultural University, School of Life Sciences, National Engineering Laboratory of Crop Stress Resistance Breeding, Hefei, China.; 2 West Anhui University, College of Biological and Pharmaceutical Engineering, Lu’an, China.

**Keywords:** Waterlogging stress, cmh15, RNA-seq, transcription factor, maize

## Abstract

Waterlogging stress is an important abiotic stress that adversely affects maize growth and yield. The mechanism regulating the early stage of the maize response to waterlogging stress is largely unknown. In this study, CM37 and cmh15 seedlings were treated with waterlogging stress and then examined in terms of their physiological changes. The results indicated that inbred line cmh15 is more tolerant to waterlogging stress and less susceptible to peroxide-based damages than CM37. The RNA sequencing analysis identified 1,359 down-regulated genes and 830 up-regulated genes in the waterlogging-treated cmh15 plants (relative to the corresponding control levels). According to the Gene Ontology analysis for the differentially expressed genes (DEGs), some important terms were identified which may play important roles in the response to waterlogging stress. Moreover, enriched Kyoto Encyclopedia of Genes and Genomes pathways were also identified for the DEGs. Furthermore, the substantial changes in the expression of 36 key transcription factors may be closely related to the maize in response to waterlogging stress. This study offers important insights into the mechanism in regulating maize tolerance to waterlogging stress, with important foundations for future research.

## Introduction

Maize (*Zea mays* L.) is a very important cereal crop cultivated globally because it is used as a source of food, feed, and fuel (Scott and Emery, 2016; Choudhary et al., 2020). Abiotic stresses, including salinity, heat, cold, drought, and waterlogging, seriously affect maize growth and development, thereby influencing the final grain quality and yield (Peng et al., 2022). Waterlogging stress significantly decreases maize yields in tropical and subtropical regions (Du et al., 2017; Yao, 2021). In recent years, global warming has resulted in frequent extreme weather events worldwide; these events have exacerbated the detrimental effects of waterlogging stress on maize (Pan et al., 2021). In areas where maize is extensively cultivated, heavy rainfall occurring over a short period can result in waterlogged soils, which can severely damage maize seedlings (Osman et al., 2013). Therefore, identifying waterlogging-responsive genes and elucidating the mechanisms underlying maize responses to waterlogging stress are essential for developing new waterlogging-tolerant maize varieties (Zaidi et al., 2004; Qiu et al., 2007; Arora et al., 2017).

Plants have evolved various strategies to withstand waterlogging stress, including morphological changes, chemical changes (e.g., redox reactions), and hormonal changes (Zhang et al., 2017). When plants are waterlogged, they undergo morphological changes that enable them to absorb oxygen and compensate for the energy loss caused by metabolic disruptions. The main morphological changes are the rapid elongation of the apical meristem tissue, the formation of adventitious roots (ARs) or other aeration tissues, barriers to radial oxygen loss, and the formation of air films in the upper cuticle (Hattori et al., 2009; Pedersen et al., 2009; Yamauchi et al., 2017; Pan et al., 2021). Through these morphological changes, plants can promote air exchange and the absorption of nutrients and water, which can stabilize the metabolic cycle and allow plants to grow normally (Steffens and Rasmussen, 2016; Qi et al., 2019).

Under waterlogging stress conditions, reactive oxygen species (ROS) contents in plants are balanced via the regulation of antioxidant enzyme systems and other active antioxidants, which helps to reduce damages caused by stress (Zhang et al., 2007; Doupis et al., 2017). Waterlogging leads to hypoxia in plant cells, which increases intracellular ROS levels, especially hydrogen peroxide (H_2_O_2_), leading to cell death and plant senescence (Bailey-Serres and Chang, 2005; Mary et al., 2012; Pucciariello et al., 2012). Nicotinamide adenine dinucleotide phosphate (NADPH) oxidase (NOX), which is primarily responsible for ROS production when plants are exposed to hypoxic conditions, plays a significant role in ROS-mediated signal transduction in plants (Pan et al., 2021). Waterlogging stress induces the expression of NOX-related gene *AtRbohD* in *Arabidopsis*, which positively regulating the production of H_2_O_2_ and enhancing the tolerance of *Arabidopsis* to waterlogging stress (Yang CY et al., 2015; Sun et al., 2018). In a previous study in which several maize varieties were treated with waterlogging stress, the waterlogging-tolerant varieties had increased peroxidase (POD), superoxide dismutase (SOD), and catalase (CAT) activities (Li et al., 2018). Similarly, a comparison of cucumber varieties exposed to waterlogging stress revealed POD, SOD, and CAT activities are lower in waterlogging-sensitive plants than in waterlogging-tolerant plants (Li, 2007). According to the findings of these earlier studies, plants that are relatively resistant to waterlogging stress typically have highly active antioxidant enzymes and ROS scavengers.

Plant hormones, such as ethylene (ETH) and abscisic acid (ABA), are critical for plant responses to waterlogging stress (Yang and Choi, 2006; Bashar, 2018; Qi et al., 2019, 2020; Hu et al., 2020). For example, the *Arabidopsis* response to hypoxic conditions involves the regulated expression of the ETH response factor (ERF) gene *ERF73/HRE1* (Hess et al., 2011; Yang, 2014). In maize, *ZmEREB180* encodes a positive regulator of AR formation and ROS levels; the overexpression of *ZmEREB180* enhances survival during prolonged periods of waterlogging stress (Yu et al., 2019). Additionally, ABA is a crucial regulator of the plant water potential and stomatal opening, especially under waterlogged conditions (Kim et al., 2021). When soybean hypocotyls are waterlogged, the ABA concentration decreases quickly and the secondary aerenchyma appears after 72 h, but the application of exogenous ABA inhibits the development of aerenchyma cells, implying ABA influences root aerenchyma development (Shimamura et al., 2014).

The identification of waterlogging-responsive genes is important for creating novel waterlogging-tolerant maize varieties. The new maize variety An’nong 876 has several excellent characteristics, including the resistance to multiple stresses (e.g., drought and heat) and high yields. In this study, comparison between cmh15 (the paternal parent of An’nong 876) and CM37 (the maternal parent of An’nong 876) seedlings exposed to waterlogging stress indicated that cmh15 is more tolerant to waterlogging than CM37. The gene expression profiles of cmh15 under the waterlogging treatment were investigated via transcriptome sequencing, and some key genes responsive to waterlogging were screened. The candidate genes identified in this study may be useful for the molecular breeding of waterlogging-tolerant maize as well as for future studies conducted to clarify the mechanism mediating the maize response to waterlogging stress.

## Material and Methods

### Plant materials and waterlogging treatment

The seeds of the cmh15 and CM37 inbred lines were provided by Professors Qing Ma and Beijiu Cheng. The seeds were sown in a greenhouse with a 16-h light (28 °C)/8-h dark (23 °C) photoperiod. At the three-leaf stage, the seedlings underwent the waterlogging treatment by adding water until the water level was 2-3 cm above the soil surface. The control seedlings were watered normally. The third leaf was collected from the waterlogging-treated and control seedlings 6 days later. They were immediately frozen in liquid nitrogen and stored at −80 °C for the subsequent RNA isolation. For the transcriptome sequencing analysis, three biological replicates were prepared for the control group (CKM-1, CKM-2, and CKM-3) and the waterlogging treatment group (WM-1, WM-2, and WM-3).

### Measurement of physiological and morphological indicators

Morphological indicators were analyzed for the plants in the treatment and control groups, including plant height, root length, fresh weight, dry weight, and the differences between two groups were determined (Qiu et al., 2007). After the 6-day waterlogging treatment, the middle part of the third leaf was collected from the waterlogging-treated and control seedlings to examine the accumulation of H_2_O_2_ via diaminobenzidine (DAB) staining chromogenic kit according to manufacturer’s instructions (Nanjing Jiancheng Bioengineering Institute, Nanjing, China).

### Construction of cDNA libraries and RNA sequencing

The TRIzol Reagent Mini Kit (Qiagen ChinaCo., Ltd, Shanghai, China) was used to extract total RNA from each leaf sample. The total RNA samples were quantified and the quality was assessed using the Agilent 2100 Bioanalyzer (Agilent Technologies, Palo Alto, CA, USA) and the NanoDrop spectrophotometer (Thermo Fisher Scientific Inc.). The cDNA libraries were prepared using 1 μg total RNA according to the manufacturer’s protocol, which involved several key steps, including mRNA fragmentation, cDNA synthesis, adapter ligation, PCR amplification, and purification. The constructed cDNA libraries with various indices were sequenced using the Illumina HiSeq system (Illumina, San Diego, CA, USA).

### Sequence assembly and data analysis

To acquire high-quality clean data, the raw data were processed using Cutadapt (v1.9.1), which removed adapters, sequences shorter than 75 bp, and low-quality sequences (Q < 20) from the 5′ and 3′ ends of the reads (Martin, 2011). The clean reads were aligned to the maize B73 reference genome (RefGen_v4) using HISAT2 (v2.0.1) (Kim et al., 2015). In addition, HTSeq (v0.6.1) was used to calculate the fragments per kilobase of exon per million mapped fragments (FPKM) value for each transcript (Anders et al., 2015). The DESeq2 (v1.6.3) Bioconductor package was used for the differential expression analysis (Anders and Huber, 2010; Anders and Huber, 2012; Love et al., 2014). The differentially expressed genes (DEGs) between the control and waterlogging treatment groups were identified using the following criteria: |log_2_(FC)| ≥ 1 and adjusted p value ≤ 0.05. The key differentially expressed transcription factors (TF) were identified according to the following criterion: |log2(FC)| > 2.

### Validation of RNA sequencing data by quantitative real-time PCR

Eight DEGs were selected for the quantitative real-time PCR (qRT-PCR) analysis to verify the accuracy of the RNA sequencing (RNA-seq) data. First-strand cDNA was synthesized from RNA using the PrimeScript RT reagent Kit with gDNA Eraser (TaKaRa, China). The Primer Premier 5 (v5.0) was used to design gene-specific primers (Table S1). The maize *GAPDH* gene (accession number: NM_001111943.1) served as an internal control for normalizing gene expression levels. The qRT-PCR analysis was performed as previously described (Zhao et al., 2019) and the 2^−ΔΔCt^ method was used to calculate relative expression levels (Livak and Schmittgen, 2001).

### Gene Ontology and pathway enrichment analyses

The DEGs were annotated by Gene Ontology (GO) analysis using GOSeq (v1.34.1) (Harris et al., 2004), which include three main functional categories (i.e., biological process, molecular function, and cellular component). The Kyoto Encyclopedia of Genes and Genomes (KEGG) database was used for pathway enrichment analysis for the identified DEGs (Kanehisa and Goto, 2000).

## Results

### Characteristics of cmh15 and CM37 under waterlogged conditions

**Figure 1 -:**
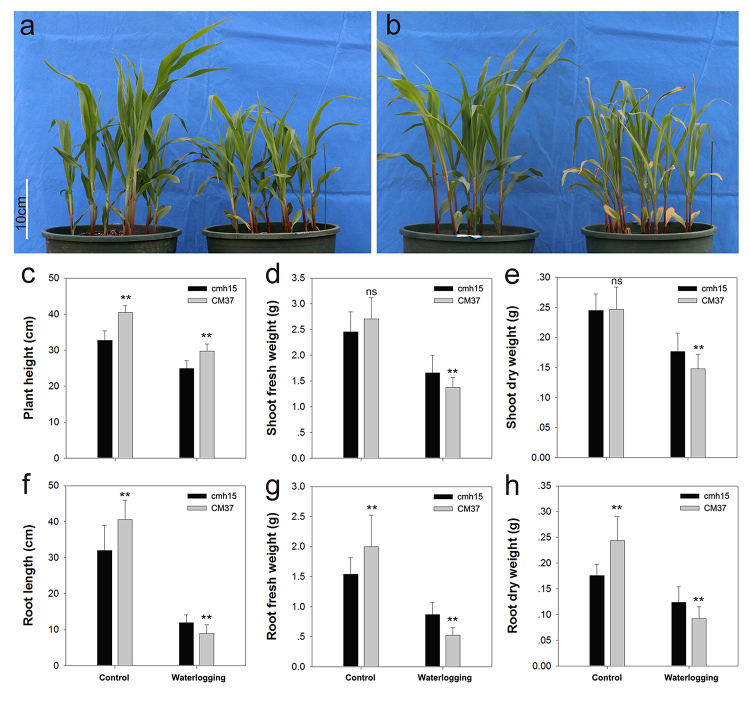
Phenotypic and physiological responses of cmh15 and CM37 seedlings. **(a)** and **(b)** Phenotypic response of cmh15 and CM37 seedlings under control and waterlogging stress conditions. Scale bar = 10 cm. **(c-h)** Plant height, shoot fresh weight, shoot dry weight, root length, root fresh weight, and root dry weight. Data represent mean values ± SE. ** p ≤ 0.01; ns, not significant (calculated by t-test).

**Figure 2 -:**
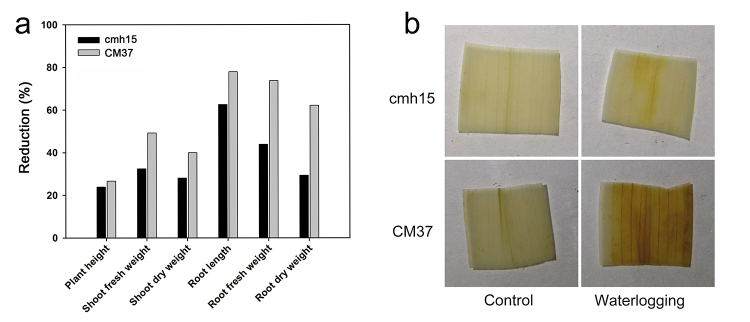
Physiological and morphological responses of cmh15 and CM37 seedlings. (**a)** Relative reduction of phenotypic indexes. **(b)** DAB staining of cmh15 and CM37 leaves.

The maize cmh15 and CM37 seedlings exposed to waterlogging stress at the three-leaf stage were examined. The results indicated that the growth of cmh15 and CM37 was significantly affected under waterlogging stress, however, compared with cmh15, the CM37 seedlings exhibited yellowing and wilting leaves and their first leaves were severely yellow (Figure 1a and b). Analysis of the plant height, root length, root fresh weight, root dry weight, shoot fresh weight, and shoot dry weight indicated the biomass loss due to waterlogging was less for cmh15 than for CM37 (Figures 1c-h, 2a). For the analysis of the accumulation of H_2_O_2_ in plant leaves via DAB staining, the CM37 leaves were more intensely stained than the cmh15 leaves, suggesting more H_2_O_2_ was accumulated in CM37 than in cmh15 (Figure 2b). Accordingly, the cmh15 seedlings appeared to be more tolerant to waterlogging stress than the CM37 seedlings.

### RNA-seq analysis of cmh15

**Figure 3 -:**
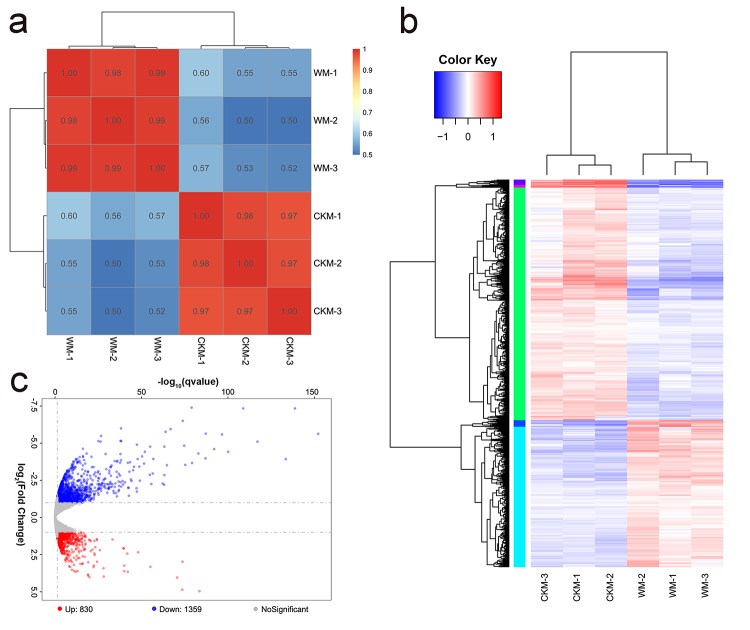
Correlation analysis and differentially expressed genes of cmh15 between control and waterlogging stress conditions. **(a)** The correlation heat map of the samples. **(b)** Heatmap analysis of the DEGs. **(c)** Volcano plot of the DEGs.

Because cmh15 was more tolerant to waterlogging stress than CM37, the RNA-seq analysis was performed using the control and waterlogging treatment groups of cmh15 to detect significant DEGs, which may include key genes involved in the response to waterlogging stress. Six samples from the cmh15 control (CKM1-CKM3) and waterlogging treatment (WM1-WM3) groups were used to construct cDNA libraries. The RNA-seq analysis of each cDNA library yielded 39.54-45.60 million raw reads. For the six libraries, 258,361,476 clean reads were retained after the raw reads were filtered for quality. Approximately 72.82%-74.58% of the clean reads were uniquely mapped to the maize B73 reference genome (Table 1). The heatmap clustering results indicated that the three biological replicates for the control and treatment groups were clustered together (Figure 3a). The principal component analysis of the six samples revealed the high correlation between the replicates of each group (Figure S1). Thus, the RNA-seq data were highly reproducible.

**Table 1 -:** Detailed information of RNA-seq data from the 6 samples.

Samples	Raw reads	Clean reads	Q20 (%)	Q30 (%)	GC (%)	Uniquely mapped (%)
CKM-1	43,941,600	43,808,848	96.09	88.10	56.48	73.62
CKM-2	45,567,336	45,430,658	96.10	88.02	55.78	74.08
CKM-3	43,819,902	43,670,054	95.75	87.20	55.72	72.82
WM-1	42,345,044	42,199,446	96.11	88.07	55.06	74.58
WM-2	39,542,668	39,415,028	96.38	88.64	54.47	74.29
WM-3	43,989,062	43,837,442	95.80	87.24	54.96	74.16

### Identification of DEGs in the response to waterlogging stress

On the basis of the statistical analysis of the expressed genes, the most common FPKM values were 3-15, whereas the least common FPKM values were > 60 (Table S2). According to the comparison of the FPKM values of all expressed genes between the control and waterlogging treatment groups, slight increase in expression was observed in the waterlogging treatment group than in the control group, indicative of the waterlogging-induced expression of some genes (Figure S2).

**Figure 4 -:**
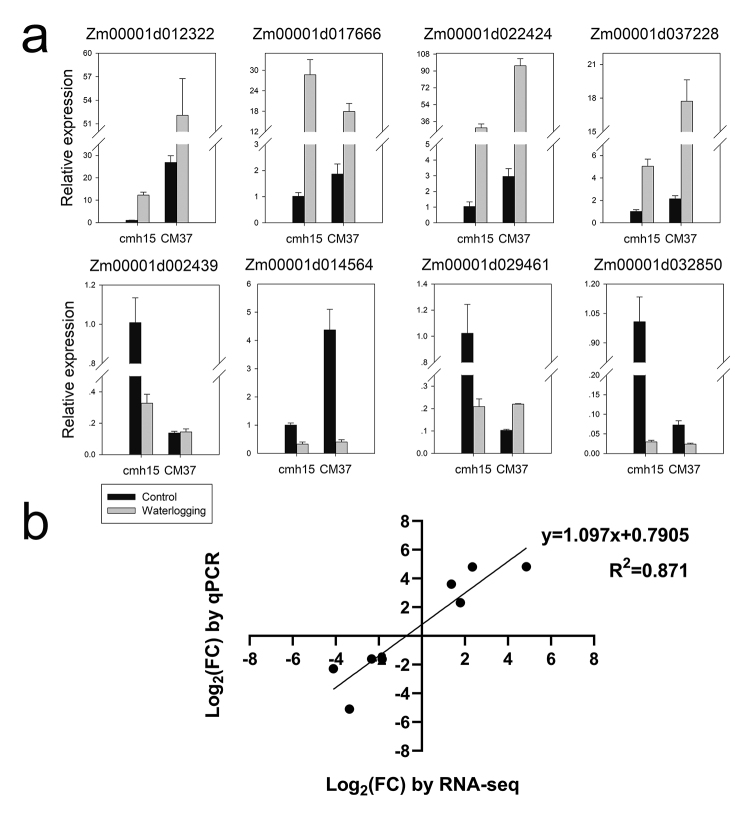
Quantitative RT-PCR validation of differentially expressed genes. **(a)** Relative expression level of 8 selected genes. **(b)** Correlation analysis of the RNA-Seq data (log2 FC) and qRT-PCR (log2 FC) for the cmh15 under control and waterlogging stress conditions.

The DEGs were screened and subjected to a cluster analysis. The three biological replicates for each group were clustered (Figure 3b). In total, 2,189 DEGs were identified between the control and waterlogging treatment groups, including 1,359 down-regulated genes and 830 up-regulated genes (Figure 3c). Four up-regulated DEGs and four down-regulated DEGs were selected for the qRT-PCR analysis. The correlation between the qRT-PCR and RNA-seq data (*R* ^*2*^ = 0.871) reflected the reliability of the RNA-seq results (Figure 4).

### Enrichment analysis of the DEGs

**Figure 5 -:**
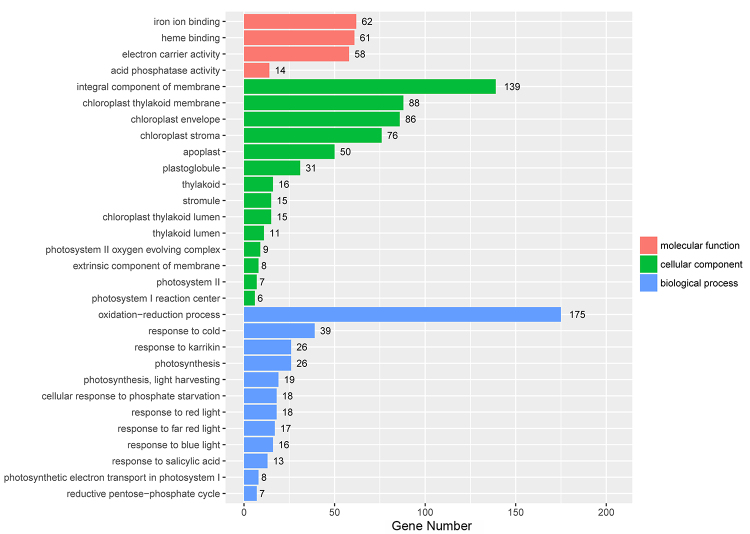
GO enrichment analysis for the 2,189 DEGs.

**Figure 6 -:**
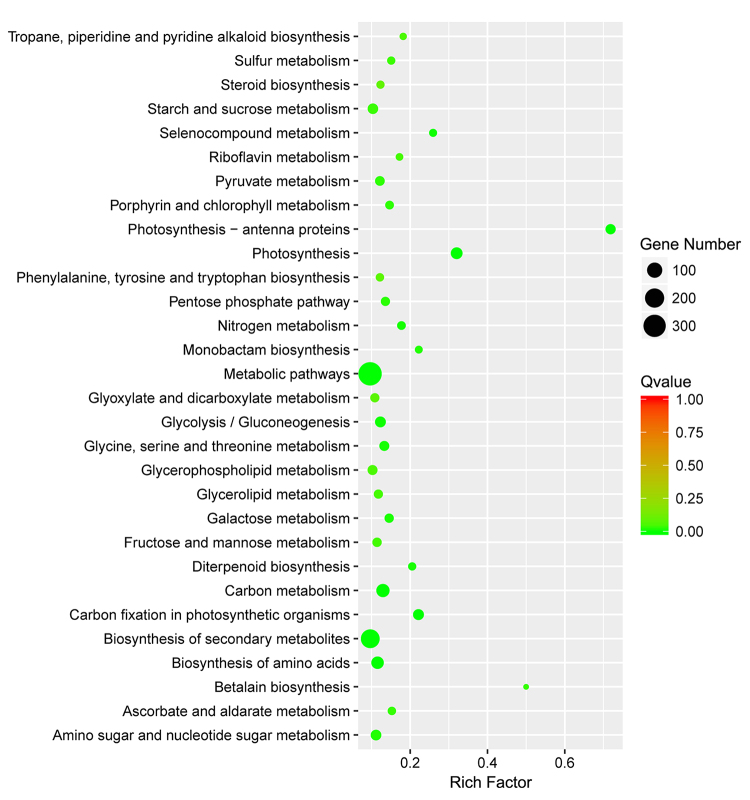
KEGG pathway enrichment analysis for the 2,189 DEGs.

To investigate the biological roles of the DEGs responsive to waterlogging stress, the 2,189 DEGs between the control and waterlogging treatment groups were functionally annotated using GO enrichment analysis. Figure 5 shows the 30 most significantly enriched GO terms. Within the molecular function category, iron ion binding (GO:0005506) and heme binding (GO:0009055) were mainly enriched. In the biological process category, oxidation-reduction process (GO:0055114) and response to cold (GO:0009409) were the main enriched GO terms. Within the cellular component category, integral component of membrane (GO:0016021) and chloroplast (GO:0009507) were mainly enriched. Some of the DEGs annotated with these terms may play a vital role in the response of maize to waterlogging stress. For example, among the genes annotated with oxidation-reduction process (GO:0055114), *Zm00001d020686* (*acco2*) is important for the final step of the ETH biosynthesis pathway (Ning et al., 2021). The KEGG analysis of these DEGs identified 121 enriched pathways. Figure 6 shows the 30 most significantly enriched pathways. Four pathways, including biosynthesis of amino acids (ko01230), metabolic pathways (ko01100), biosynthesis of secondary metabolites (ko01110) and carbon metabolism (ko01200), were enriched with the highest number of DEGs. Additionally, other pathways, such as glycolysis/gluconeogenesis (ko00010), may be closely related to the waterlogging stress response of maize.

### Analysis of key DEGs encoding TFs

**Figure 7 -:**
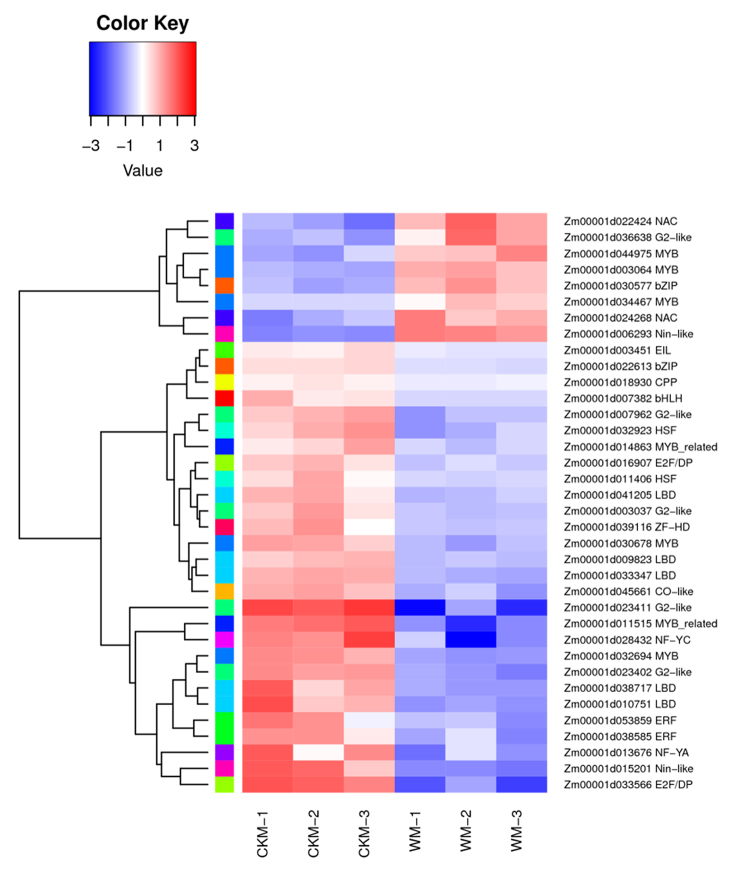
Heatmap analysis of the 36 key transcription factors.

A total of 155 TFs from 37 TF families were identified from the DEGs. The families with the most TFs were MYB, G2-like, and bZIP. On the basis of the RNA-seq analysis, 36 of the 155 TFs were detected as the key differentially expressed members (|log_2_(FC)| > 2). The cluster analysis of these 36 TFs showed that eight TFs had up-regulated expression levels in response to the waterlogging treatment, whereas the expression levels of the other 28 TFs were down-regulated (Figure 7). Due to the limited number of genes reported in response to waterlogging stress in maize, the analysis of the homologs of these TFs in *Arabidopsis* and rice suggested that some TFs may be important for regulating the maize response to abiotic stress and hormone responses. For example, a previous study showed that the overexpression of *OMTN6*, which is the rice homolog of *Zm00001d024268*, negatively affects the drought resistance of rice in the reproductive stage (Fang et al., 2014).

## Discussion

Waterlogging is one of the important factors limiting global maize production. In the summer maize planting area in the Huang-Huai-Hai region, which accounts for more than one-third of the entire maize planting area in China, more than two-thirds of the annual precipitation is concentrated in the summer maize growing period (Wang et al., 2022). Excessive rainfall may result in waterlogged soils, which can seriously affect the summer maize yield and quality (Ren et al., 2015). There have been some researches on the mechanism mediating waterlogging tolerance (Pan et al., 2021). In fact, multiple response mechanisms have been established in plant response to waterlogging stress, one of the responsive mechanisms of waterlogging stress is the expression changes of many genes, thus, identification of the key responsive genes can play an important foundation for the related mechanism research.

There are relevant studies indicating that at the first two days during the onset of waterlogging, the waterlogging tolerance coefficient (WTCs) decreased slowly, but it started to show sharp decline from days 4 to 6, and the trend of descent became very slightly from days 8 to 12 (Liu et al., 2010). According to related studies, a period of 6-10 days is commonly employed for treating waterlogging stress (Du et al., 2016; Ren et al., 2017; Huang et al, 2022). In addition, different germplasm backgrounds have different responses to waterlogging stress. Therefore, we chose 6 days as the waterlogging treatment time in the experiments according to related studies, which also can reflect the responsive differences between cmh15 and CM37. The results showed that the CM37 leaves were more wilted and yellowed compared with cmh15 in waterlogging groups (Figure 1a and 1b). Analysis of some indicators such as plant height indicated that the biomass loss was more for CM37 than for cmh15 under waterlogging treatment (Figures 1 and 2a). For the analysis of the accumulation of H_2_O_2_ in plant leaves via DAB staining, we found that CM37 accumulated more H_2_O_2_ than cmh15 after waterlogging treatment (Figure 2b). Combined with the analysis of these phenotypic and physiological indicators, we concluded that cmh15 was more tolerant to waterlogging stress than CM37. The control and waterlogging treatment groups of cmh15 were included in the RNA-seq analysis performed in this study, and 1,359 down-regulated and 830 up-regulated DEGs were identified. The mainly enriched GO terms for the DEGs were oxidation-reduction process (GO:0055114) and integral component of membrane (GO:0016021). Furthermore, DEGs were enriched in some GO terms related to photosynthesis, such as photosynthesis, light harvesting (GO:0009765), photosystem I reaction center (GO:0009538) and photosystem II (GO:0009523). According to previous studies, waterlogging can inhibit the activity of photosynthesis related enzymes (Voesenek et al., 2006; Wu and Yang, 2016). The KEGG pathway enrichment analysis indicated that metabolic pathways (ko01100) were highly enriched for the DEGs in the cmh15 waterlogging treatment group (Figure 6). In a previous study, the RNA-seq analysis of the waterlogging-tolerant maize line ‘Suwan-2’ revealed metabolic pathways was significantly enriched, which may be related to waterlogging tolerance (Yao, 2021). Furthermore, the KEGG pathway analysis indicated glycolysis/gluconeogenesis (ko00010) was an enriched pathway among the DEGs. An earlier study determined that plants exposed to hypoxia due to waterlogging can continue to produce energy to a certain extent through glycolysis and ethanol fermentation (Pan et al., 2021). Therefore, the findings suggested the important roles of these terms and pathways in response to waterlogging stress.

Transcription factors play a vital role in plant responses to abiotic stresses and hormones (Wang et al., 2018). The *ZmEREB180* encodes a TF that regulates waterlogging tolerance in maize seedlings by enhancing AR formation and antioxidant levels (Yu et al., 2019). According to the RNA-seq analysis, 155 differentially expressed TFs were identified, while 36 had significant changes in their expression levels in response to waterlogging stress (|log_2_FC| > 2). The analysis of these TFs and their homologs in *Arabidopsis* and rice revealed the importance of some TFs in response to abiotic stress and hormone responses. Due to ETH diffusion rate in water being low, waterlogging stress resulted in the accumulation of ETH in plant tissues, which will induce the expression of genes involved in the response to waterlogging stress (Voesenek and Bailey-Serres, 2015). Furthermore, ETH can regulate the formation of the plant aerenchyma and ARs, while also controlling the elongation of branches to cope with waterlogging stress. Earlier research showed the homolog of *Zm00001d003451* (*EIL5*) in rice (*OsEIL6*) affects ETH signal transduction in rice plants (Mao et al., 2006; Yang C et al., 2015). Because the ability to regulate the plant water potential, ABA is considered another key hormone in waterlogging stress (Pan et al., 2021). The homologs of *Zm00001d032923* (*HSF30*) and *Zm00001d044975* (*c1*) in rice and *Arabidopsis* are involved in ABA signal transduction (Huang et al., 2016; Wang *et al.*, 2020). Therefore, the TFs encoded by these genes may have important regulate roles in plant responses to waterlogging stress.

In conclusion, the maize response to waterlogging stress involves many complex biological processes. The findings of this study are important for breeding waterlogging-tolerant maize varieties, while also serving as the basis for future research on the responsive mechanisms to waterlogging stress.

## Supplementary material

The following online material is available for this article:

Figure S1 - Principal component analysis of the samples of the cmh15 seedlings under control and waterlogging stress conditions.

Figure S2 - Comparison of gene expression levels under control and waterlogging treatment conditions. (a) FPKM distribution of the samples. (b) FPKM box plot of the samples.

Table S1 - Gene specific primers used for qRT PCR analysis.

Table S2 - Statistics of genes in different expression level interval.
