# Supplementary material for: Identification and characterization of waterlogging-responsive genes in the parental line of maize hybrid An’nong 876
Source: Genet Mol Biol. 2024 Jan 8;46(4):e20230026. doi: 10.1590/1678-4685-GMB-2023-0026 (PMC10789244; doi:10.1590/1678-4685-GMB-2023-0026)
Supplement: Figure S1 - [file 1415-4757-GMB-46-4-e20230026-s1.pdf]

Genetics and Molecular Biology
https://doi.org/10.1590/1678-4685-GMB-2023-0026

Supplementary Material to "Identification and characterization of waterlogging-responsive genes in the parental line of maize hybrid An'nong 876"

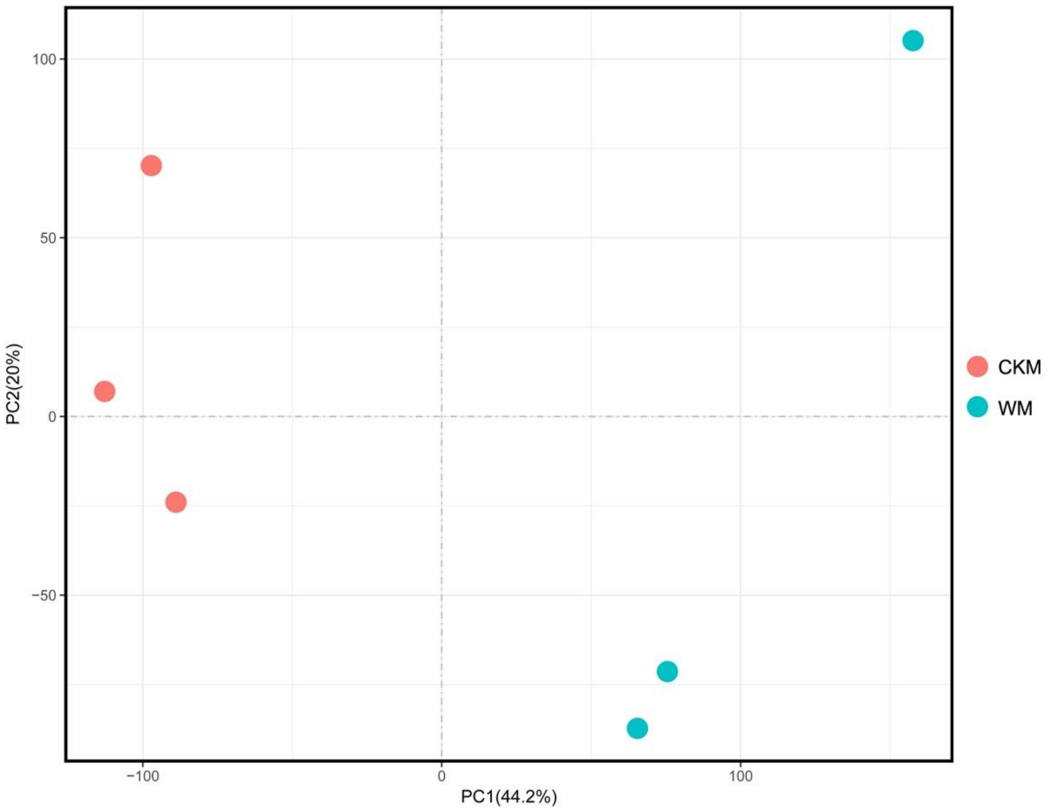

Figure S1 - Principal component analysis of the samples of the cmh15 seedlings under control and waterlogging stress conditions.
