# Supplementary material for: Identification and characterization of waterlogging-responsive genes in the parental line of maize hybrid An’nong 876
Source: Genet Mol Biol. 2024 Jan 8;46(4):e20230026. doi: 10.1590/1678-4685-GMB-2023-0026 (PMC10789244; doi:10.1590/1678-4685-GMB-2023-0026)
Supplement: Figure S2 - [file 1415-4757-GMB-46-4-e20230026-s2.pdf]

Genetics and Molecular Biology
https://doi.org/10.1590/1678-4685-GMB-2023-0026

Supplementary Material to "Identification and characterization of waterlogging-responsive genes in the parental line of maize hybrid An'nong 876"

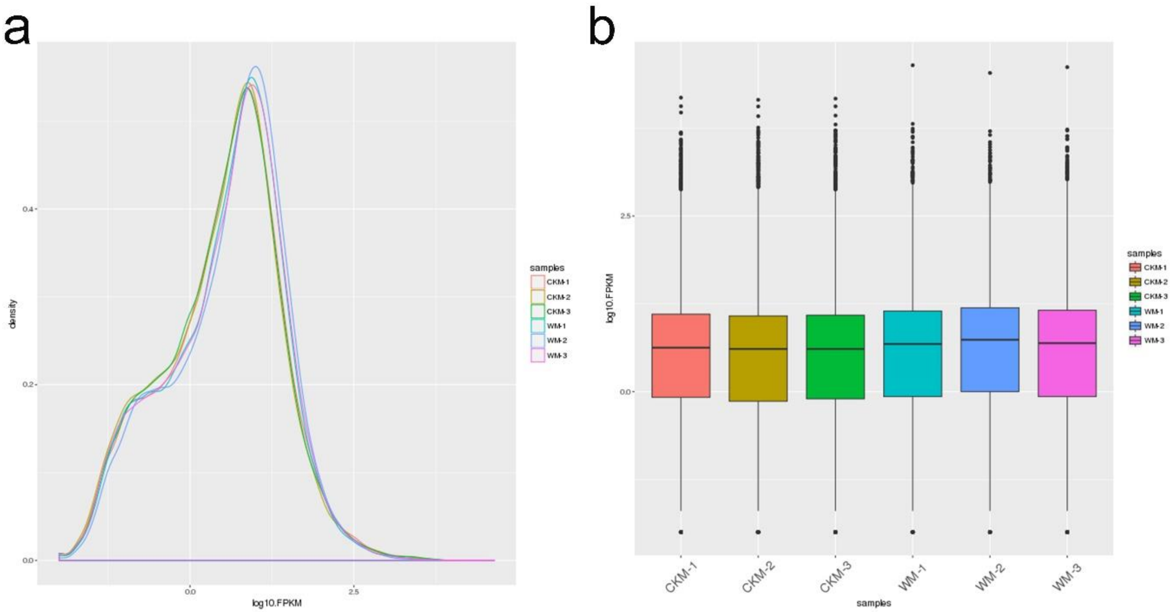

Figure S2 - Comparison of gene expression levels under control and waterlogging treatment conditions. (a) FPKM distribution of the samples. (b) FPKM box plot of the samples.
