# Supplementary material for: Identification and characterization of waterlogging-responsive genes in the parental line of maize hybrid An’nong 876
Source: Genet Mol Biol. 2024 Jan 8;46(4):e20230026. doi: 10.1590/1678-4685-GMB-2023-0026 (PMC10789244; doi:10.1590/1678-4685-GMB-2023-0026)
Supplement: Table S1 - [file 1415-4757-GMB-46-4-e20230026-s3.pdf]

Genetics and Molecular Biology

https://doi.org/10.1590/1678-4685-GMB-2023-0026

Supplementary Material to "Identification and characterization of waterlogging-responsive genes in the parental line of maize hybrid An'nong 876"

Table S1 - Gene-specific primers used for qRT-PCR analysis.

**Gene name**	**Primer 5´-->3´**
GAPDH-F	ATCAACGGCTTCGGAAGGAT
GAPDH-R	CCGTGGACGGTGTCGTACTT
Zm00001d012322-F	ACACGGACACCTTTATCAAC
Zm00001d012322-R	ACGTTCGACTCGATCTCTT
Zm00001d037228-F	TAGGGGATCGATCAGATATCCA
Zm00001d037228-R	GATCTCGTTCGAGTAGTTCACC
Zm00001d017666-F	CTCTGCACCATCCTCTACTC
Zm00001d017666-R	GACTGTGAGAATGTCGAAGG
Zm00001d022424-F	GGCTTCTTCGACGACTACTT
Zm00001d022424-R	GGCGTCGTAGTAGTGTTTGT
Zm00001d029461-F	GATCATAGCTGTGCTGTTCA
Zm00001d029461-R	CAAAGGCTTCGTAACAATTC
Zm00001d014564-F	GAAGGAGAACATCAAGAACG
Zm00001d014564-R	CTCGAATACGCCAATGAC
Zm00001d002439-F	GCCACCTCCAGAAGTATAGA
Zm00001d002439-R	CTCTTGTGTTCTTCCCTTGA
Zm00001d032850-F	CACCTTCTTCTTCGCAAACTTC
Zm00001d032850-R	GAGGATAAAGCCAAGCAAGTTG
