# Supplementary material for: Identification and characterization of waterlogging-responsive genes in the parental line of maize hybrid An’nong 876
Source: Genet Mol Biol. 2024 Jan 8;46(4):e20230026. doi: 10.1590/1678-4685-GMB-2023-0026 (PMC10789244; doi:10.1590/1678-4685-GMB-2023-0026)
Supplement: Table S2 - [file 1415-4757-GMB-46-4-e20230026-s4.pdf]

Genetics and Molecular Biology

https://doi.org/10.1590/1678-4685-GMB-2023-0026

Supplementary Material to "Identification and characterization of waterlogging-responsive genes in the parental line of maize hybrid An'nong 876"

Table S2 - Statistics of genes in different expression-level interval.

Samples	FPKM 0-0.1	FPKM 0.1-1	FPKM 1-3	FPKM 3-15	FPKM 15-60	FPKM > 60
CKM-1	1,821 (7.13%)	5,113 (20.01%)	4,116 (16.31%)	8,926 (34.94%)	4,072 (15.94%)	1,448 (5.67%)
CKM-2	1,952 (7.71%)	5,284 (20.86%)	4,046 (15.97%)	8,889 (35.10%)	3,839 (15.16%)	1,318 (5.20%)
CKM-3	1,791 (7.07%)	5,306 (20.96%)	4,073 (16.09%)	8,837 (34.91%)	3,942 (15.57%)	1,368 (5.40%)
WM-1	1,799 (7.03%)	5,041 (19.69%)	3,780 (14.77%)	8,957 (34.99%)	4,691 (18.33%)	1,330 (5.20%)
WM-2	1,527 (5.84%)	5,014 (19.18%)	3,607 (13.80%)	9,232 (35.31%)	5,322 (20.35%)	1,445 (5.53%)
WM-3	1,867 (7.33%)	4,965 (19.50%)	3,701 (14.53%)	8,782 (34.48%)	4,762 (18.70%)	1,390 (5.46%)
